# Opening the Policy Window to Mobilize Action Against Corruption in the Health Sector: Comment on "We Need to Talk About Corruption in Health Systems"

**DOI:** 10.15171/ijhpm.2019.65

**Published:** 2019-08-07

**Authors:** Tim K. Mackey

**Affiliations:** ^1^Global Health Policy Institute, San Diego, CA, USA.; ^2^Department of Anesthesiology and Division of Infectious Diseases and Global Public Health, School of Medicine, University of California, San Diego, CA, USA.

**Keywords:** Corruption, Health Corruption, Public Policy, Global Health Governance, Transparency and Accountability

## Abstract

Corruption in the health sector has been a "dirty secret" in the health policy and international development community, but recent global activities point to a day when it will no longer be neglected as a key determinant of health. To further explore next steps forward, this commentary applies the Kingdon’s multiple-streams framework (MSF) to assess what opportunities are available to mobilize the global agenda to combat health corruption. Based on this analysis, it appears that Kingdon’s problem, policy, and political streams are coalescing to create a policy window opportunity that can be leveraged based on recent developments in the global health and international development community around corruption. This includes the recent formation of the Global Network on Anti-Corruption, Transparency and Accountability (GNACTA) led by the World Health Organization (WHO), the Global Fund, and the United Nations Development Programme in 2019. It also includes bridging shared goals of addressing corruption in order to make progress towards health-specific goals in the United Nations (UN) Sustainable Development Goals (SDGs) and for achieving universal health coverage.

Corruption in the health system has indeed been a “dirty secret” in health policy and international development circles as mentioned in the article “We Need to Talk About Corruption in Health Systems” by Hutchinson and colleagues.^1^ However, health corruption, while still “dirty,” is becoming less “secret,” with a growing number of studies explicitly linking the estimated $300 billion US dollars lost yearly to health sector corruption to poor health outcomes, faltering health systems and even patient death.^2-5^ In this sense, the various manifestations of health corruption are no longer unknown – we now know and recognize that it is a major barrier to shared goals of sustainable development, health equity, and achieving universal health coverage.^6,7^ The challenge now is moving the fight against corruption to the next phase, which means putting in the hard work of cultivating the necessary partnerships, generating data, developing tools, and mobilizing political will to detect, characterize, prevent, and enforce against corruption as a key priority for the future of global health.

Reflecting some of these challenges, Hutchinson et aldescribe their five reasons explaining why corruption has yet to break through in the international health policy discourse. Many of these challenges are familiar to anti-corruption experts including: lack of a concrete definition for corruption (particularly given the various types and forms of corruption and its cultural context), the need to address the real-world practicalities of why corruption exists in fragile health systems (eg, the use of informal payments to access healthcare services), difficulties in conducting empirical research on health corruption practices (particularly in the context of lack of transparency, accountability and difficulty in collecting corruption-related data), and the challenging ethical barriers of researching, implementing, and evaluating anti-corruption activities, particularly when the subjects of said corrupt activities (including individual actors involved in sporadic or petty corruption up to policy-makers/politicians involved in institutional and political corruption) are complicit, benefit, or are directly involved in the corruption itself.^1,8,9^

Authors also ask the fundamental question of whether it is legitimate to study corruption, while also highlighting findings from a 2016 Cochrane review by Gaitonde et al that found limited evidence for the effectiveness of anti-corruption strategies.^10^ While these observations are valid concerns, it does not take away from the fact that the study of health corruption is not just legitimate but is now being recognized by a number of different international stakeholders as crucial to the current and future success of global health programs and initiatives as outlined below.^5^

Authors conclude that there is a need to convene key stakeholders to debate, reach consensus, and develop an international agenda to empower people to talk about corruption in health systems. Here, authors should be encouraged that such activities are already underway and have new and exciting support from the international community. Specifically, this includes the recent formation of the Global Network on Anti-Corruption, Transparency and Accountability (GNACTA) led by the World Health Organization (WHO), the Global Fund, and the United Nations Development Programme in 2019. As key organizations representing constituencies in the international public health, multilateral global health funding, and international development spheres, GNACTA is positioned to act as a key health anti-corruption fora now and into the future provided it receives appropriate support and a strong mandate to take action. Hutchinson et al also suggest the need to prioritize actions based on risk to vulnerable groups and impact on health systems. This is a logical conclusion, but one that is already being carried out in the field through risk-based fraud, auditing, and law enforcement practices.^5,7^ Hence, efforts are well underway, but the journey to adequately control health sector corruption will be continuous as corruption simply cannot be eradicated like an infectious disease, instead it is chronic to healthcare systems.

In response, this commentary focuses not on the limitations of tackling health sector corruption, but the opportunities to leverage existing international momentum by adopting John Kingdon’s multiple-streams framework (MSF) to identify priorities needed to address this seminal global health challenge.^11^ The piece focuses on applying MSF to the examination of existing policy, programmatic, and governance frameworks, such as GNACTA (https://hsgovcollab.org/en/event/consultation-proposed-global-network-anti-corruption-transparency-and-accountability-health), the United Nations (UN) Sustainable Development Goals (SDGs) (https://sustainabledevelopment.un.org/sdgs), and the UN Convention against Corruption (UNCAC) (https://www.unodc.org/unodc/en/treaties/CAC/), and how they can form a unique convergence event for a “policy window” to mobilize anti-corruption global health governance.

## Kindgon’sPolicy Window for Health Anti-corruption?

It has been over 30 years since John Kingdon first published the MSF in his book “Agendas, Alternatives and Public Policies,” which originally focused on public policy agenda setting in the US domestic policy context.^11^ However, Kingdon’s MSF continues to hold relevance today, including in the area of comparative policy analysis and health systems research.^12^ Simply put, MSF examines agenda setting processes focusing on three “streams” including the “problem stream,” “policy stream,” and the “political stream” that primarily act independently. However, when these streams converge, they create an opportunity for a “policy window” that can help facilitate policy change.^12^ Below we describe each of these streams and how they can create a global policy window specific to mobilizing international anti-corruption efforts in the health sector.

The first MSF stream is the problem stream, which begins with recognition of a public problem that requires government or state action for resolution. The problem stream requires elevating attention to public matters so they can be recognized and acted upon by decision-makers (eg, policy-makers and political leaders). Garnering attention or political pressure to a problem often requires dramatic events that raise awareness to the issue simultaneously among the public and policy-makers audiences. When politicians or government official decision-makers themselves are directly implicated in corruption, this is particularly challenging, often requiring public outrage, civil society mobilization, anti-corruption advocacy campaigns (eg, letter writing and online campaigns, petitions, earned and traditional media, etc), and even litigation in the hopes of mobilizing anti-corruption coalitions or creating pressure for government/regime change or anti-corruption policy-making.

From a macro historical level, taking corruption out of the shadows and elevating it to the problem stream has occurred through a progression of important voices that have helped de-stigmatize the word “corruption.” This includes the seminal 1996 speech by former World Bank President James Wolfensohn’s, calling for global action against the “cancer of corruption,” to more recent statements by former World Bank President Jim Yong Kim, who denounced corruption as “public enemy number one,” and former US Secretary of State John Kerry, who characterized corruption as a “pandemic.”^7^

Further, high-profile events such as allegations of corruption and misuse of funds in the Global Fund’s portfolio reported by the Associated Press in 2011 have raised alarms across the global health and international development aid communities, as well as rebuke from donors.^7,13^ This event, though viewed as damaging to overall global health prospects, can also be seen as a critical juncture in bringing needed recognition that the presence of corruption, no matter the size (as reports of the $34 million in missing funds for this incident were less than 1% of the Global Fund’s total grant portfolio) has the power to undermine the integrity of multibillion dollar global health programs, hence elevating the issue to the problem stream.^7^ Public awareness and fear of health corruption has also appeared in domestic survey data where the Kaiser Family Foundation found that corruption is viewed as the biggest barrier to improving health in developing countries among US respondents.^5^ This is particularly important as the United States remains the top source for development assistance for health.^14^

Second, the MSF policy stream involves the conceptualization and proposal of solutions to address identified problems. More simply, if a problem is identified but has no policy solution, then it cannot be acted upon or included in the decision-making agenda.^11^ Hence, decision-makers will prioritize problems that already have needed solutions in place, though those decision-makers involved in corruption (whether they be democratically elected representatives, technocrats, or non-democratic regimes) themselves may either block, amend, or refocus solutions to externalize the problem based on their own preferences, particularly in the case of systemic corruption or state capture. Hence, there is a need for a broad base of proposed solutions that can be generated by experts (including academia, civil society, and other stakeholders) and can then be independently assessed for action or inaction, narrowed to those that represent feasible policy change options, and then championed by a broad coalition of anti-corruption constituents to pressure political action and accountability. Here, though evidence to support the effectiveness of anti-corruption measures remains relatively weak, the necessary collection of experts that can create multisector solutions to address health corruption is rapidly taking shape. GNACTA represents such a convening place to generate diverse anti-corruption solution options that can be designed in conjunction with existing national law and internationally binding legal instruments coupled with multistakeholder advocacy and technical assistance to countries.

Existing domestic and international policy instruments that can be leveraged as solutions include UNCAC, a universal anti-corruption instrument that focuses on prevention, criminalization, international cooperation, asset recovery, and technical assistance and information exchange, which can be specifically tailored to corruption in the health sector.^9^ It also includes domestic anti-corruption laws, such as the US Foreign Corrupt Practices Act and the UK Bribery Act, whose influence could be extended by adoption of similar laws and regulations in other countries. Critically, health corruption is a trans-sectoral issue, particularly in the context of how corruption negatively impacts the social determinants of health and health equity. Hence, anti-corruption approaches cannot simply be limited to the health sector and instead should be focused on trans-sectoral interventions in other industrial, economic, and social spaces. Legal, treaty, and governance instruments such as UNCAC, the US Foreign Corrupt Practices Act, the UK Bribery Act, and the SDGs represent optimal convening places to tackle corruption from this trans-sectoral approach.

The third “politics stream” is made up of factors that are political in nature (eg, changes in governments/administrations, upswing in public opinion, and increased advocacy/lobbying) that can ostensibly generate the political will needed to take action against a problem. Here again, GNACTA’s formation represents a critical opportunity for mobilizing both partnership but also multistakeholder advocacy around combating health corruption. Collectively, GNACTA enjoys the participation of members from international organizations, multilateral development agencies, academia, national government officials, and civil society, all of whom can collectively advocate to their national governments and the UN system under a unified voice and multistakeholder platform. Bolstering GNACTA is also the UN SDGs, which via Goals 3 and 16 provide evidence of global consensus (including specific SDG targets and indicators) that improving health *and* combating corruption are key pillars for 21st century development.^6^

Hence, the converge of the MSF “problem,” “policy,” and “political” streams around health corruption appear to be taking place. Specifically, the “problem” of health corruption is now evident in the increased number of publications around the subject (including a special collection of health corruption articles currently being developed with partnership from the WHO) and recognition of the problem through civil society advocacy and international meetings such as the 2016 Anti-Corruption Summit held by former UK Prime Minister David Cameroon. The “policy stream” is also maturing with domestic and international anti-corruption instruments to enforce against corruption and global governance approaches included in the SDGs to measure health and corruption-related indicators as a measure of progress towards sustainable development. Finally, the “political” stream is also emerging, with recognition of the unique dangers of health corruption among various international organizations personified by GNACTA, but also included at the recent G20 Osaka Summit where the topics of global health and anti-corruption were both included in the G20 Osaka Leaders’ Declaration.^15^

Lastly, the rise of autocratic regimes in certain countries has raised the profile of corruption among the broader public. This includes high-income countries such as the United States, where public perception and concerns about corruption in government and the executive branch are becoming more pronounced.^16,17^ Hence, signs are pointing to greater public awareness and fear of the influence of corruption, potentially unlocking the final stream of public awareness that can force the “opening” of a health corruption Kingdon policy window.

## Conclusion

As the streams of problem, policy, and politics converge around corruption, a policy window appears to be materializing that can take us to the next phase of fighting corruption in health. Further, beyond policy and governance-focused approaches, specific anti-corruption solutions, such as targeted risk assessment using a heat map to prioritize anti-corruption interventions for issues with the highest likelihood and negative impact, evaluation frameworks that trace impact on financial, performance, and health indicators, and good governance tools (such as the WHO Good Governance for Medicines tool), are already in place and ready to be utilized.^18^ Another important solution area are new technology applications that could represent next generation solutions, powered by innovations in data mining, machine learning, and emerging technologies such as blockchain.^7,19^ In fact, during the inaugural GNACTA meeting in Geneva, Switzerland, an “Experience Exchange Marketplace” was held where select participants shared their research and results from anti-corruption solutions and case studies providing examples of health anti-corruption in practice. Importantly, establishing a research agenda to empirically test these solutions is necessary to support evidence-based policy-making and implementation in the field, though that should not delay the maturing of a robust “policy” and “solution” stream for health corruption.

The success of GNACTA will be critical to opening Kingdon’s policy window and walking through it. However, I am already encouraged by progression made since my first publication on health corruption in 2012, when reviewers from certain journals commented that corruption was important, but we in the global health community should not talk about it as it could negatively impact all the great work being done in global health. Now, that attitude has shifted with anti-corruption research being encouraged, meetings and open debate supported by international organizations, and the word “corruption” in the health sector no longer being whispered.

## Ethical issues

Not applicable.

## Competing interests

Author declares that he has no competing interests.

## Author’s contribution

TKM is the single author of the paper.
